# Effect of anatomical liver resection for hepatocellular carcinoma: a systematic review and meta-analysis

**DOI:** 10.1097/JS9.0000000000000503

**Published:** 2023-05-26

**Authors:** Seong Wook Shin, Tae-Seok Kim, Keun Soo Ahn, Yong Hoon Kim, Koo Jeong Kang

**Affiliations:** a Division of Transplantation and Vascular Surgery; b Division of Hepatobiliary Surgery, Department of Surgery, Dongsan Medical Center, Keimyung University School of Medicine, Daegu, Korea

**Keywords:** anatomical liver resection, hepatocellular carcinoma, liver resection, non-anatomical liver resection

## Abstract

**Background:**

Despite retrospective studies comparing anatomical liver resection (AR) and non-anatomical liver resection (NAR), the efficacy and benefits of AR for hepatocellular carcinoma remain unclear.

**Materials and methods:**

The authors systemically reviewed MEDLINE, Embase, and Cochrane Library for propensity score matched cohort studies that compared AR and NAR for hepatocellular carcinoma. Primary outcomes were overall survival (OS) and recurrence-free survival (RFS). Secondary outcomes were recurrence patterns and perioperative outcomes.

**Results:**

Overall, 22 propensity score matched studies (AR, *n*=2,496; NAR, *n*=2590) were included. AR including systemic segmentectomy was superior to NAR regarding the 3-year and 5-year OS. AR showed significantly better 1-year, 3-year, and 5-year RFS than NAR, with low local and multiple intrahepatic recurrence rates. In the subgroup analyses of tumour diameter less than or equal to 5 cm and tumours with microscopic spread, the RFS in the AR group was significantly better than that in the NAR group. Patients with cirrhotic liver in the AR group showed comparable 3-year and 5-year RFS with the NAR group. Postoperative overall complications were comparable between AR and NAR.

**Conclusions:**

This meta-analysis demonstrated that AR showed better OS and RFS with a low local and multiple intra-hepatic recurrence rate than NAR, especially in patients with tumour diameter less than or equal to 5 cm and non-cirrhotic liver.

## Introduction

Highlights

- The superiority of anatomical liver resection over non-anatomical liver resection for hepatocellular carcinoma remains controversial.
- We conducted systematic review and meta-analysis including 22 propensity score-matched cohort studies.
- This meta-analysis demonstrated that anatomical liver resection showed better overall survival and recurrence free survival with a low local and multiple intrahepatic recurrence rate than non-anatomical liver resection, especially in patients with tumour diameter less than or equal to 5 cm and non-cirrhotic liver.

Hepatocellular carcinoma (HCC) accounts for ~90% of primary liver cancer. According to the clinical practice guideline of the European Association for the Study of the Liver (EASL), liver resection and liver transplantation were recommended as the first options in patients with very-early (single <2 cm, preserved liver function, PS 0) and early-stage (single or 2–3 nodules <3 cm, preserved liver function, PS 0) tumours^1^. With the shortage of liver transplantation donors, liver resection is the mainstay of HCC treatment. Advancing surgical techniques including minimally invasive liver resection (laparoscopic or robotic liver resection) and perioperative management, postoperative mortalities and severe postoperative morbidities have been decreasing in experienced centres. However, the high 5-year recurrence rate after liver resection, roughly estimated up to 80%, is a huge challenge for long-term prognosis^2^. Approximately 80–90% of postoperative recurrences were intrahepatic recurrences. Intrahepatic recurrence could be multicentric occurrence (de novo HCC) or intrahepatic metastasis. Intrahepatic metastasis is the main cause of HCC recurrence in the residual liver within 2 years postoperatively and is the consequence of subclinical metastases from primary tumour growth through microvascular invasion (MVI)^3^. Theoretically, anatomic liver resection (AR), which involves the systemic removal of the tumour bearing portal territories with exposure of the landmark veins framing the segmental territory, has been thought to be effective and beneficial for eradicating the intrahepatic metastases of HCC. Although some studies demonstrated the superiority of AR over non-anatomical liver resection (NAR), others revealed conflicting results^4–7^. Owing to the lack of large-scale randomized controlled trials^8^, most studies were retrospective ones. The contradictory results on AR for HCC might be attributed to the comparison of non-homogeneous cohorts including the tendency to perform wider resection in patients with well-preserved liver function. Moreover, across studies, some heterogeneities regarding patient inclusion criteria and definition of AR exist.

To minimize these biases in comparing oncologic outcomes between two surgical methods in retrospective studies, we analyzed propensity score matching (PSM) studies in the present study. The aim of this meta-analysis to compare true oncologic outcomes of AR and NAR for HCC treatment with current best available evidence. Also, we compared the oncologic outcomes of two surgical methods through subgroup analysis to investigate how characteristics such as tumour size, MVI, cirrhosis, and segmentectomy are associated with the intervention effects.

## Materials and methods

This systematic review was conducted following the guidelines for the Preferred Reporting Items for Systematic Reviews and Meta-analyses (PRISMA, Supplemental Digital Content 1, http://links.lww.com/JS9/A586, Supplemental Digital Content 2, http://links.lww.com/JS9/A587) 2020 and AMSTAR-2 guidelines^9,10^, Supplemental Digital Content 3, http://links.lww.com/JS9/A588.

### Criteria for considering studies in this review and registration

#### Type of studies

Because only one randomized controlled trial is performed^11^, we included PSM studies irrespective of language to minimize confounders.

#### Type of participants

We included patients diagnosed with primary HCC without extrahepatic metastasis and macrovascular invasion. We excluded patients who previously received anticancer or adjuvant therapies, such as radiofrequency ablation, transcatheter arterial chemoembolization, percutaneous ethanol injection, and chemotherapy.

#### Types of interventions

We set NAR as the comparator and AR as an intervention.

#### Type of outcomes measures

The primary outcomes were overall survival (OS) and recurrence-free survival (RFS). Secondary outcomes were recurrence patterns (local, intrahepatic, and extrahepatic recurrences) and perioperative outcomes (overall complications).

#### Registration

We registered our protocol with PROSPERO.

### Search methods for the identification of studies

#### Searching strategy

We systemically searched Medline, Embase, and Cochrane Library for PSM studies published up to 1 August 2022. The initial database search was performed on 23 April 2020. The last database search was undertaken on 10 August 2022. A search strategy (Supplementary material 1, Supplemental Digital Content 4, http://links.lww.com/JS9/A592) for this systemic review was conducted for each database using a combination of Medical Subject Heading and text words.

#### Searching other resources

Also, we reviewed the reference list of the identified articles.

### Data collection and analysis

#### Selection of studies

Two independent reviewers assessed 90 articles by screening their titles and abstracts. Then, two reviewers screened full-text articles. After excluding 68 articles (18 non-PSM studies, 1 randomized controlled trial, 1 PSM study including patients with recurrent tumours, 1 PSM study including patients with MVI, 1 PSM study including patients with macroscopic bile duct tumour thrombosis, 16 conference abstracts, 5 studies including patients who received anticancer treatment preoperatively, 3 studies including patients received adjuvant therapies, 3 studies including patients with non-primary HCC, 17 review articles, and 2 articles with unavailable full texts), the remaining 22 PSM studies were included in the final analysis.

#### Data extraction and assessment of the risk of bias (RoB)

After reviewing 22 full-text articles, three reviewers extracted the following data: number of patients, demographics (age and sex), liver viral status (viral hepatitis B or C), liver function status (i.e. serum albumin, serum bilirubin, platelet count, cirrhosis, Child–Pugh class, and Model for End-Stage Liver Disease score), and tumour factor (i.e. diameter, number, location, MVI, satellite nodule, and tumour differentiation). The primary outcomes were 1-year, 3-year, and 5-year OS and RFS. Hazard ratio (HR) was used as the effect size of time-to-event data (i.e. OS and RFS) with 95% CI as described by Tierney *et al.* ^12^. Next, secondary outcomes were perioperative outcomes (i.e. overall complications, bile leakage, hepatic failure, blood loss, operative time, and postoperative hospital stay) and recurrence patterns (i.e. local recurrence).

We used the Risk Of Bias In Non-randomized Studies of Intervention tool for assessing the RoB in included studies regarding biases in confounding, selecting participants into the study, classification of intervention, deviations from intended interventions, missing data, measuring outcomes, and selecting reported results^13^. Rovis was used as a visualizing tool (http://www.riskofbias.info)^14^.

### Statistical analysis

We compared the OS and RFS using log HR and their log standard error. Log odds ratio for comparing recurrence pattern (i.e. local recurrence or single/multiple recurrence), and perioperative outcomes (i.e., overall complication, bile leakage, and postoperative hepatic failure) were also computed within studies. We used the generic inverse–variance method with a random-effects model for the meta-analysis, in which weight was given to each study according to the inverse of the variance of the effect to minimize uncertainty about the pooled effect estimates. The magnitude of statistical heterogeneity was assessed by $I^{2}$ statistic. Subgroup analyses were performed to explore possible causes of heterogeneity. Additionally, sensitivity analyses were carried out by sequentially removing individual studies to evaluate the robustness of the pooled results of these studies. All tests of statistical significance were two-sided, and significance was set at *P*=0.05. Most statistical analyses were performed using Review Manager software version 5.4 (The Cochrane Collaboration). Sensitivity analysis and Egger’s regression test were performed using R version 3.6.2 (https://cran.r-project.org).

## Results

### Included studies

**Figure 1:**
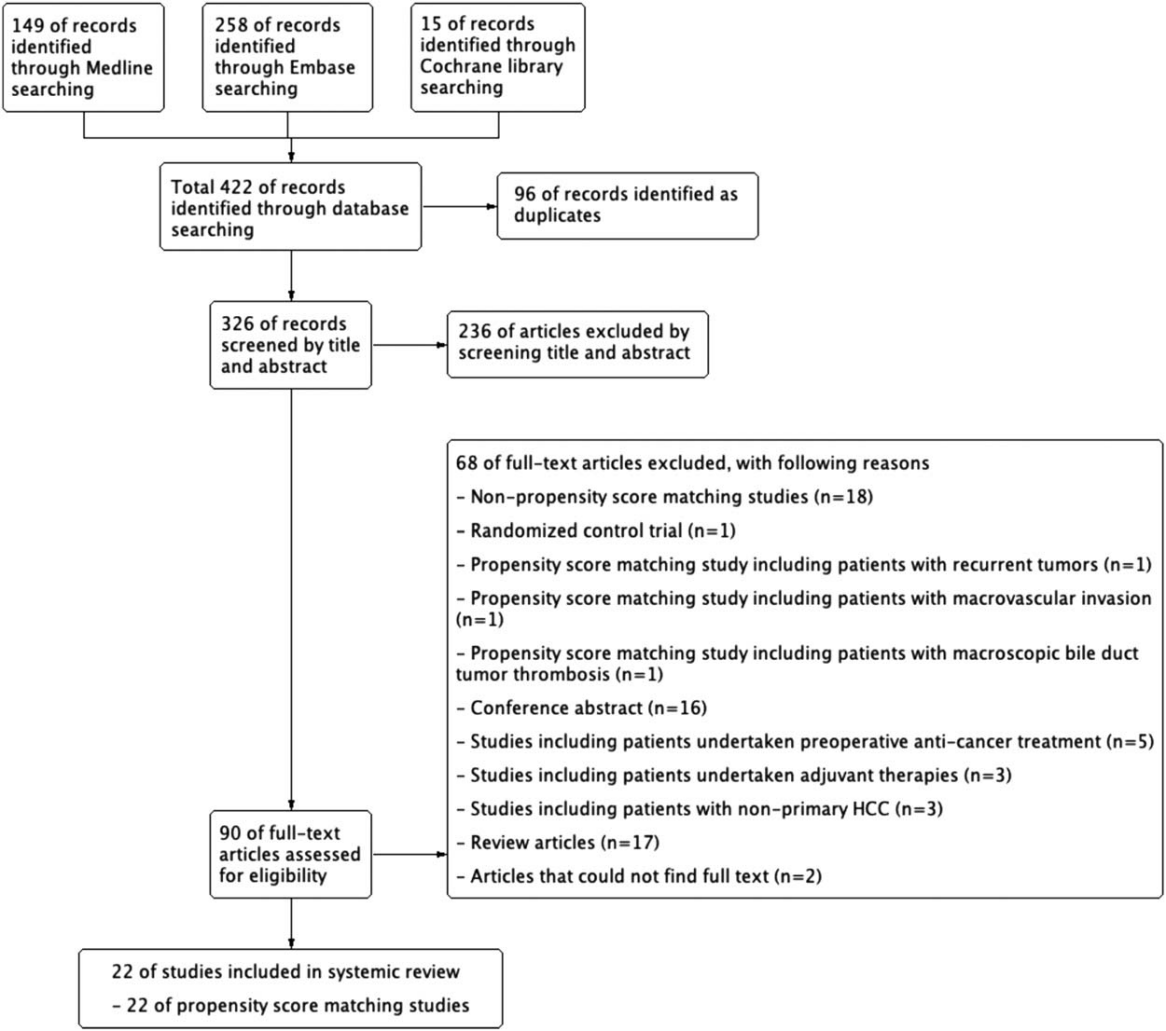
Flow diagram showing the selection of studies. HCC, hepatocellular carcinoma.

Overall, 326 studies were identified after excluding duplicates. Of these, we excluded 236 articles by screening titles and abstracts. The remaining 90 studies were included for full-text review. After excluding 68 studies (Fig. 1), 22 PSM studies^4,6,15–34^ were finally included in this study.

A total of 5,086 patients (AR, *n* = 2,496; NAR, *n*=2,590) were enroled. Their characteristics are shown in Table 1. Twelve of the studies were conducted in Japan (54.5%), five in China (22.7%), three in Italy (13.6%), two in Korea (9.1%), and one each in Spain and Taiwan. Patients’ median or mean age ranged from 46.9 to 71 years. Most patients were male (up to 90%). Many patients presented with viral hepatitis, and the proportion of causative viral disease varied in each region. The proportion of hepatitis B infection tended to be high in Korea and China, whereas that of hepatitis C infection tended to be high in Japan and Italy. In each study, 18–100% of the patients presented with underlying liver cirrhosis, and at least 92% were of Child–Pugh class A. Twelve studies (54.5%) included only patients with solitary HCC, and the proportion of patients with solitary HCC was also high (77–96%) in the remaining nine studies. The median or mean tumour diameter ranges from 2.3 to 6 cm.

**Table 1:** List of included studies.

Studies	Year	Country	Type of design	Inclusion criteria	Definition of AR	No. patients (AR/NAR)
Cho *et al*.^6^	2019	Korea	Retrospective, PSM	Patients with solitary HCC with tumour size <5 cm	Including segmentectomy	59/59
Cucchetti^15^	2013	Italy	Retrospective, PSM	Patients HCC within the Milan criteria and with liver cirrhosis	Including segmentectomy	149/149
Famularo^16^ (1)	2018	Italy	Retrospective, PSM	Patients with histologically proven HCC	Including segmentectomy	177/177
Famularo^17^ (2)	2018	Italy	Retrospective, PSM	Patients with histologically proven HCC and liver cirrhosis	Including segmentectomy	100/100
Haruki *et al*.^18^	2021	Japan	Retrospective, PSM	Primary HCCs ≤ 5 cm	Including segmentectomy	66/66
Hidaka *et al*.^19^	2020	Japan	Retrospective, PSM	Patients with solitary HCC and microportal invasion (Vp1)	Including segmentectomy	86/86
Hirokawa *et al*.^20^	2015	Japan	Retrospective, PSM	Patients with solitary HCC and tumour size <5 cm	Including segmentectomy	72/72
Huang *et al*.^21^	2015	China	Retrospective, PSM	Patients with histologically proven HCC	Including segmentectomy	278/278
Huang*et al*.^4^	2020	Taiwan, Japan	Retrospective, PSM	Patients with HCC and tumour size*<*5 cm.	Including segmentectomy	76/76
Ishii *et al*.^22^	2014	Japan	Retrospective, PSM	Patients with histologically HCC	Including segmentectomy	44/44
Kaibori *et al*.^23^ (1)	2020	Japan, Korea	Retrospective, PSM	Patients with solitary HCC and tumour size*<*5 cm	Excluding segmentectomy	355/355
Kaibori *et al*.^24^ (2)	2020	Japan	Retrospective, PSM	Patients with solitary HCC and tumour size < 3 cm	Only systemic segmentectomy	114/114
Kitano *et al*.^25^	2022	Japan	Retrospective, PSM	Primary HCC within the Milan criteria	Including segmentectomy	210/210
Minagawa *et al*.^26^	2021	Japan	Retrospective, PSM	Patients with solitary HCC and tumour size*<*5 cm	Only systemic segmentectomy	67/67
Molina-Romero *et al*.^27^	2019	Spain	Retrospective, PSM	Patients with primary HCC and Child–Pugh class A	Including segmentectomy	17/17
Okamura *et al*.^28^	2014	Japan	Retrospective, PSM	Patients with solitary HCC	Including segmentectomy	64/64
Shindoh *et al*.^29^ (1)	2016	Japan	Retrospective, PSM	Patients with solitary HCC and tumour size*<*5 cm and Child–Pugh class A and ICG-R15< 30%	Only systemic segmentectomy	156/53
Shindoh *et al*.^30^ (2)	2020	Japan	Retrospective, PSM	Patients with solitary HCC and tumour size <5 cm confined to one Couinaud’s segment and Child–Pugh class A and ICG-R15*<*35%	Only systemic segmentectomy	38/165
Xu *et al*.^31^	2020	China	Retrospective, PSM	Patients with primary HCC and tumour size of 5.0–10.0 cm without macrovascular invasion	Including segmentectomy	51/51
Zhao *et al*.^32^ (1)	2017	China	Retrospective, PSM	Patients with solitary HCC	Including segmentectomy	114/114
Zhao *et al*.^33^ (2)	2020	China	Retrospective, PSM	Patients with solitary HCC and Child–Pugh class A	Including segmentectomy	103/103
Zhong *et al*.^34^	2019	China	Retrospective, PSM	Patients with primary HCC, MVI, and Child–Pugh class A	Including segmentectomy	100/170

AR, anatomical liver resection; HCC, hepatocellular carcinoma; MVI, microscopic vascular invasi; NAR, non-anatomical liver resection; PSM, propensity score matching.

### RoB in the included studies

The overall RoB is summarized in Supplementary material 2, Supplemental Digital Content 5, http://links.lww.com/JS9/A595. Regarding bias due to missing data, 14 studies demonstrated a serious risk because of unwritten information about missing or completeness of data. The remaining eight studies appropriately addressed data completeness. For bias due to confounding, most of the included studies except one study (Minagawa *et al.* ^26^) demonstrated a moderate risk because most of the expected confounders were appropriately measured and controlled by PSM. In the study of Minagawa *et al*.^26^, a higher platelet count was found in the AR group even after PSM. Regarding the bias due to the selection of the reported results, 21 studies demonstrated moderate risk without clear evidence that all reported results corresponded to all intended outcomes, analyses, and subcohorts through examination of a pre-registered protocol. However, the study by Hirokawa *et al.* ^20^ demonstrated a serious RoB, as it only compared clinicopathological characteristics of subgroups according to tumour size (<3 cm and 3–5 cm) without reporting survival outcomes.

Across these studies, some biases were noted. First, the entire cohort of studies was not homogeneous because of the different inclusion criteria (i.e. tumour size, tumour number, vascular invasion, viral hepatitis, and background liver status). Second, the definitions of AR and NAR were different between studies. The most controversial was whether to consider segmentectomy as AR or not. In the NAR group, various resection methods were included, from enucleation to wide resection with sufficient margin. Despite these biases, some systemic reviews analyzed pooled outcomes without subgroup analysis considering heterogeneity. In the present systemic review, to determine the true effect of AR on HCC, we conducted a subgroup analysis according to the tumour size and definition of surgical methods.

### OS: AR vs. NAR

**Figure 2:**
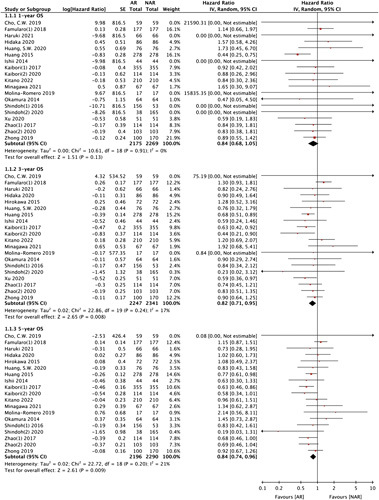
1-year, 3-year, and 5-year overall survival between AR and NAR. AR, anatomical liver resection; NAR, non-anatomical liver resection; OS, overall survival.

Twenty studies compared the OS rate between AR and NAR, and two studies^15,17^ did not provide OS information. No significant difference in the 1-year OS was found between AR and NAR. However, AR was superior to NAR in terms of 3-year and 5-year OS using random-effects model (3-year OS HR: 0.82, 95% CI: 0.71–0.95, *P*=0.008; 5-year OS HR: 0.84, 95% CI: 0.74–0.96, *P*=0.009) (Fig. 2).

### RFS: AR vs. NAR

**Figure 3:**
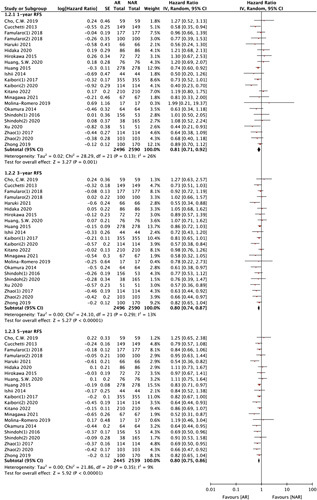
1-year, 3-year, and 5-year recurrence-free survival between AR and NAR. AR, anatomical liver resection; NAR, non-anatomical liver resection; RFS, recurrence-free survival.

All 22 studies were included in the RFS analysis. AR showed significantly better results than NAR regarding 1-year, 3-year, and 5-year RFS (1-year RFS HR: 0.81, 95% CI: 0.71–0.92, *P*=0.001; 3-year RFS HR: 0.80, 95% CI: 0.74–0.87, *P<*0.00001; 5-year RFS HR: 0.80, 95% CI: 0.75–0.86, *P<*0.00001) (Fig. 3).

### Subgroup analysis by tumour size

To evaluate the influence of surgical methods according to the tumour size, tumour size was divided into less than or equal to 5 cm and greater than 5 cm for the subgroup analysis.

#### Tumour size less than 5 cm: OS and RFS

Fourteen studies were included for the OS and RFS analyses in patients with tumour size less than 5 cm. Although no significant difference was noted in the 1-year OS and RFS between AR and NAR, AR showed superior in 3-year and 5-year OS and RFS to NAR (Supplementary material 3, Supplemental Digital Content 6, http://links.lww.com/JS9/A596).

#### Tumour size greater than 5 cm: OS and RFS

Only two studies^19,31^ demonstrated information regarding tumour size greater than 5 cm, and they were included for OS and RFS analyses of patients with tumour size greater than 5 cm. AR showed significantly superior 3-year OS and RFS to NAR (Supplementary material 4, Supplemental Digital Content 7, http://links.lww.com/JS9/A598).

### Subgroup analysis: MVI or not

**Figure 4:**
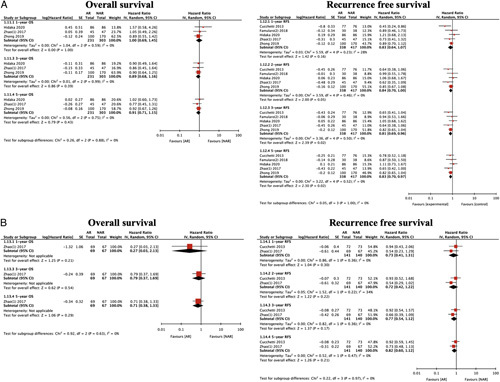
1-year, 3-year, and 5-year overall and recurrence-free survivals in subgroup analyses for patients with/without microvascular invasion. AR, anatomical liver resection; MVI, microvascular invasion; NAR, non-anatomical liver resection; OS, overall survival; RFS, recurrence-free survival.

Five studies^15,17,19,32,34^ reported the influence of surgical methods according to MVI. AR showed superior 2-year, 3-year, and 5-year RFS to NAR in patients with MVI (2-year RFS; HR 0.84, 95% CI: 0.70–1.00, *P*=0.0*5*, I ^2^=0%, 3-year RFS; HR 0.81, 95% CI: 0.69–0.96, *P*=0.02, I ^2^=0%, 5-year RFS; HR 0.83, 95% CI: 0.70–0.97, *P*=0.02, I ^2^=0%) (Fig. 4 A). However, only two studies reported the influence of surgical methods in patients without MVI, and AR was comparable to NAR in RFS (Fig. 4 B). AR failed to show better OS than NAR regardless of the presence or absence of MVI.

### Subgroup analysis: underlying liver cirrhosis or not

Four studies^15–17,21^ reported underlying liver cirrhosis. The presence or absence of cirrhosis was defined in the literature as a case confirmed on histologic specimens or clinically by a hepatologist. Although AR showed significantly better RFS than NAR in early outcomes (1 year), AR showed comparable long-term outcomes (5 years) with NAR (Supplemental Digital Contents 5a, Supplemental Digital Content 8, http://links.lww.com/JS9/A599). Even if NAR demonstrated 1-year OS superior to AR, no significant difference was found in the long-term outcomes (Supplementary material 5a, Supplemental Digital Content 8, http://links.lww.com/JS9/A599). Despite including one study^21^ that reported patients without underlying liver cirrhosis, AR demonstrated 1-year, 3-year, and 5-year OS and RFS superior to NAR (Supplementary material 5b, Supplemental Digital Content 8, http://links.lww.com/JS9/A599).

### Subgroup analysis: Segmentectomy vs. NAR

Segmentectomy was defined as the complete removal of one Couinaud’s segment or a combination of contiguous territories of the “third-order” subsegmental portal venous branches smaller than one Couinaud’s segment^35^. Four studies^24,26,29,30^ compared segmentectomy and NAR. Segmentectomy showed better 3-year and 5-year OS and RFS than NAR (Supplementary material 6, Supplemental Digital Content 9, http://links.lww.com/JS9/A600).

### Recurrence pattern

**Figure 5:**
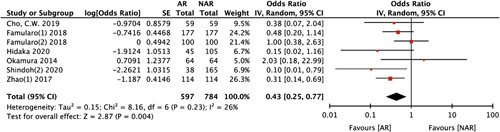
Local recurrence between anatomical liver resection (AR) and non-anatomical liver resection (NAR).

In the analysis of recurrence pattern after liver resection, the incidence of intrahepatic and extrahepatic recurrence was significantly lower in the AR group (intrahepatic recurrence: HR 0.70, 95% CI: 0.54–0.92, *P*=0.009, I ^2^=57%; extrahepatic recurrence: HR 0.74, 95% CI: 0.55–0.99, *P*=0.04, I ^2^=0%) (Supplementary material 7, Supplemental Digital Content 10, http://links.lww.com/JS9/A601). Local recurrence was defined as any recurrence in the residual part of the tumour bearing third-order portal branches after NAR or recurrence adjacent to the liver surface on initial tumour recurrence (marginal recurrence). Compared with NAR, AR significantly decreased the local recurrence rate (HR 0.43, 95% CI: 0.25–0.77, *P*=0.004, I ^2^=26%) (Fig. 5).

#### Intrahepatic single or multiple nodule recurrences

**Figure 6:**
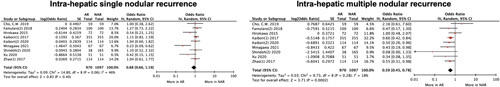
Intrahepatic single or multiple nodule recurrences between anatomical liver resection (AR) and non-anatomical liver resection (NAR).

Next, regarding the initial recurrences, no difference was found between AR and NAR for solitary intrahepatic recurrences. However, the number of patients with multiple intrahepatic recurrences in the AR group was significantly smaller than that in the NAR group (HR 0.59, 95% CI: 0.45–0.78, *P*=0.0002, I ^2^=18%) (Fig. 6).

### Overall complications including postoperative hepatic failure and biliary leakage

No significant difference in overall complications was found between the two treatment arms. The number of biliary leakage and hepatic failure cases did not differ between the AR and NAR groups (Supplementary material 8, 9, Supplemental Digital Content 11, http://links.lww.com/JS9/A602, Supplemental Digital Content 12, http://links.lww.com/JS9/A603).

## Discussion

This systematic review evaluated the true effect of AR for HCC in comparison with NAR and factors that would influence AR. Many studies reported superior or comparable outcomes of AR and NAR. The conclusion remains still controversial. Moreover, previously published systematic reviews simply compared OS and RFS between AR and NAR without specific subgroup analysis^36,37^.

Three main reasons were found for the heterogeneity in each study and across studies. Thus, the controversy of the superiority of AR has been continued in previously published systematic reviews. First, AR may have been performed more frequently in patients with well-preserved liver function than NAR. Because of the differences in background liver function between the two treatment groups, a simple comparison of OS and RFS in the entire cohort could involve a selection bias. Second, simply pooling the effect size for determining the real prognostic benefit of AR is not reasonable because the inclusion criteria regarding the tumour stage varied across the included studies, such as tumour size, number of tumours, and presence of vascular invasion or not. Third, no standardized technique or definition of AR and NAR was found. One of the controversial issues about AR is whether segmentectomy or sub-segmentectomy could be included in AR or not. Because existence of communicating portal branches between segments, especially segments VII and VIII made it technically difficult to perform the complete removal of the tumour bearing the third-order portal region. Another issue is whether the inclusion of hepatectomies larger than Couinaud’s segmentectomy would overestimate the prognostic effect of AR. According to Minagawa *et al.* ^26^, the inclusion of major hepatectomy may pose a risk for overestimation of the prognostic effect of AR because a large hepatectomy removes a greater amount of the liver parenchyma in which recurrences may occur. For these reasons, we included only PSM studies to minimize the selection bias and conducted subgroup analyses according to the tumour size, presence of underlying liver cirrhosis, and presence of MVI. Also, to evaluate the effectiveness of segmentectomy, we tried to compare segmentectomy separately with NAR.

In our systematic review, AR showed significantly better OS and RFS than NAR. Some other systematic review articles concluded that AR was significantly better than NAR in terms of RFS but not OS^38,39^. The controversial results are likely to be caused by study selection. Unlike the systematic review by Famularo *et al.* ^38^, we excluded two large-scale PSM studies that reported no significant difference in the OS between the two treatment groups, with high weight because of the following reasons. The study by Marubashi *et al*.^40^ was excluded because it included patients with recurrent HCC in the entire cohort, with an unknown proportion. The study by Li *et al.* ^41^ was also excluded due to the inclusion of patients with macrovascular invasion. We thought that the inclusion of recurrent nodules and patients with macrovascular invasion might not be appropriate for comparing the two surgical methods and demonstrating different outcomes. Our systematic review showed that AR demonstrated oncologic benefit in decreasing intrahepatic and extrahepatic recurrences. With good local control ability, AR showed significantly lower local recurrences than NAR. The solitary intrahepatic recurrence rates were comparable between the two groups; however, multiple intrahepatic recurrences occurred less frequently in the AR group. By reducing the multiple intrahepatic recurrence rates in the AR group, AR might demonstrate an oncologic advantage in the future treatment course. Therefore, AR might be able to reduce the incurable stage of HCC recurrence, which is thought to contribute to the improvement of OS. In patients with MVI, AR showed better results from early recurrence, whereas in patients without MVI, AR did not demonstrate a significant difference between the two treatment groups. These results suggest that complete removal of the tumour bearing the third-order portal territories through AR significantly reduced the intrahepatic metastasis of HCC from portal venous invasion.

In the present systematic review, patients with cirrhosis showed significantly better 1-year RFS in the AR group. However, AR demonstrated comparable 3-year and 5-year RFS with NAR. In early recurrences, the control of intrahepatic metastasis by AR gave dominant results to RFS, but eventually, as multicentric neocarcinogenesis prevailed over time, the difference in RFS disappeared.

Tumour size is also an important influencing factor to recurrence and survival. In our meta-analysis, AR showed superiority over NAR in the 3-year and 5-year RFS in patients with tumour size less than 5 cm. In patients with tumour size greater than 5 cm, AR was significantly superior to NAR only in the 3-year RFS. This means that in early recurrence, AR may demonstrate superior RFS, but in the end, the influence of tumour factors increases; eventually, no difference will be found in the long-term outcomes between the two treatment groups. To analyze the effect of segmentectomy, we performed subgroup analysis including studies^24,26,29,30^ that conducted sufficient surgical quality control. Segmentectomy significantly improved RFS in 3 and 5 years.

Also, we conducted sensitivity analyses in OS and RFS. Because of the paucity of data, we did not perform sensitivity analyses for the subgroup analyses. In the sensitivity analysis, no significant change was found in the results, which could demonstrate the robustness of the superiority of AR in OS and RFS. We used Egger’s regression test and funnel plot to assess publication bias. In Egger’s regression test and funnel plot about OS and RFS, no significant asymmetry was found (Supplementary material 10, Supplemental Digital Content 13, http://links.lww.com/JS9/A605).

This meta-analysis demonstrates some limitations. First, to eliminate selection bias, we only included retrospective PSM studies. Since this does not mean that we considered all the unexpected confounding variables, we cannot say that no selection bias is found. Thus, we analyzed outcomes using a random-effects model. Second, despite the sufficient number of studies included, the target populations all varied between studies. Thus, more detailed analyses were possible through subgroup analyses. In addition, to collect more data, we contacted the authors. Despite such our efforts, in some cases, we could not obtain all information. More sufficient evidence was not accumulated in the subgroup analysis. Third, excluding Kaibori *et al* ^23^, the rest of the studies included segmentectomy, and whether complete segmentectomy was really performed in the aspect of surgical quality control is questionable.

In conclusion, AR provided better results than NAR in not only OS but also RFS, with a low local recurrence rate, especially in patients with a tumour diameter less than 5 cm and non-cirrhotic liver.

## Ethical approval

None. Because this study is systematic review and meta-analysis, it based on aggregate data and does not involve humans or animal data. So ethical approval is not required.

## Sources of funding

This research did not receive any specific grant from funding agencies in the public, commercial, or not-for-profit sectors.

## Author contribution

T.-S.K., S.W.S., K.S.A.: conceptualization, methodology. S.W.S.: data curation, writing—original draft preparation. S.W.S., K.S.A.: software, formal analysis. T.-S.K., Y.H.K., K.J.K.: writing—review and editing, supervision.

## Conflicts of interest disclosure

The authors declare that they have no competing interests.

## Research registration Unique Identifying number (IUN)

1. Name of the registry: PROSPERO International prospective register of systemic reviews.
2. Unique Identifying number of registration ID: CRD42021225667.
3. Hyperlink to your specific registration (must be publicly accessible and will be checked):https://www.crd.york.ac.uk/prospero/display_record.php?RecordID=225667.

## Guarantor

Tae-Seok Kim is the Guarantor of this article.

## Data statement

This study is systematic review and meta-analysis, so the data statement is not available.

## Provenance and peer review

Not commissioned, externally peer-reviewed.

## Supplementary Material

SUPPLEMENTARY MATERIAL
