# Supplementary figures and images for: Effect of anatomical liver resection for hepatocellular carcinoma: a systematic review and meta-analysis
Source: Int J Surg. 2023 May 26;109(9):2784–93. doi: 10.1097/JS9.0000000000000503 (PMC10498869; doi:10.1097/JS9.0000000000000503)

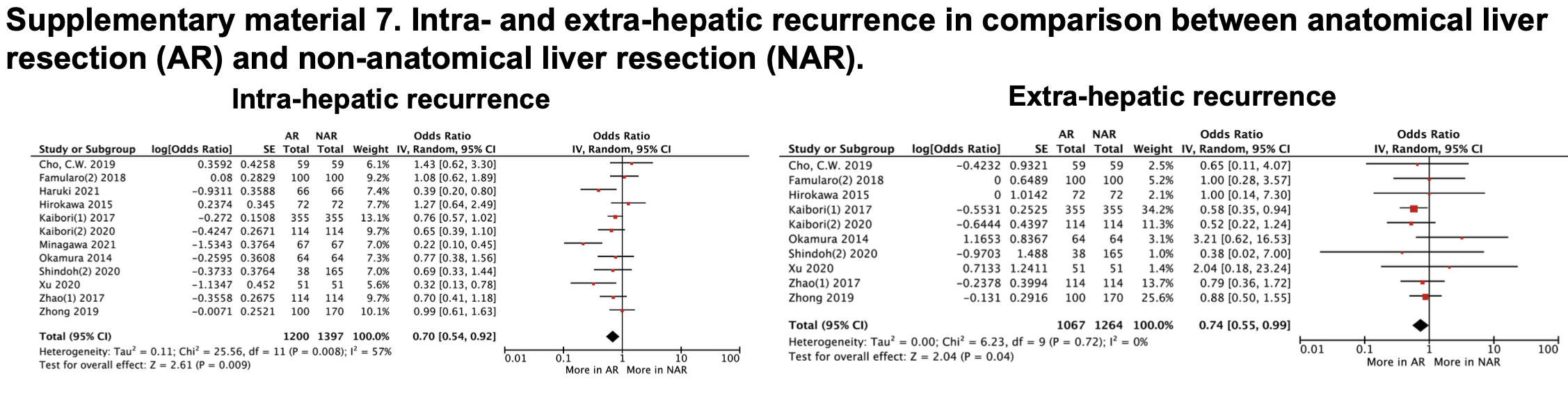

Supplement: SUPPLEMENTARY MATERIAL [file js9-109-2784-s002.jpg]

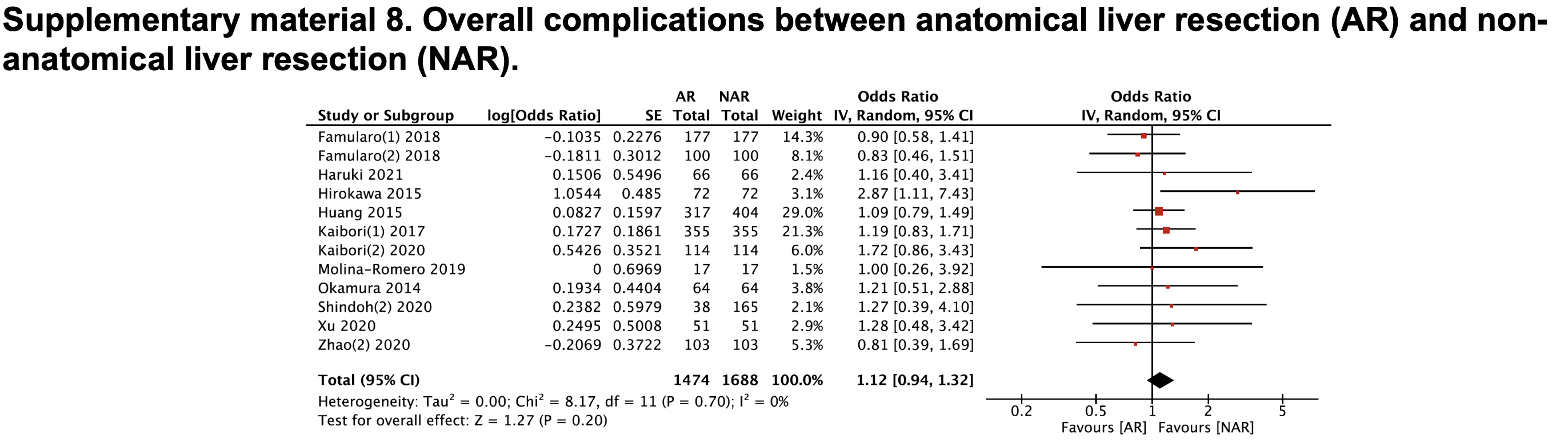

Supplement: SUPPLEMENTARY MATERIAL [file js9-109-2784-s003.jpg]

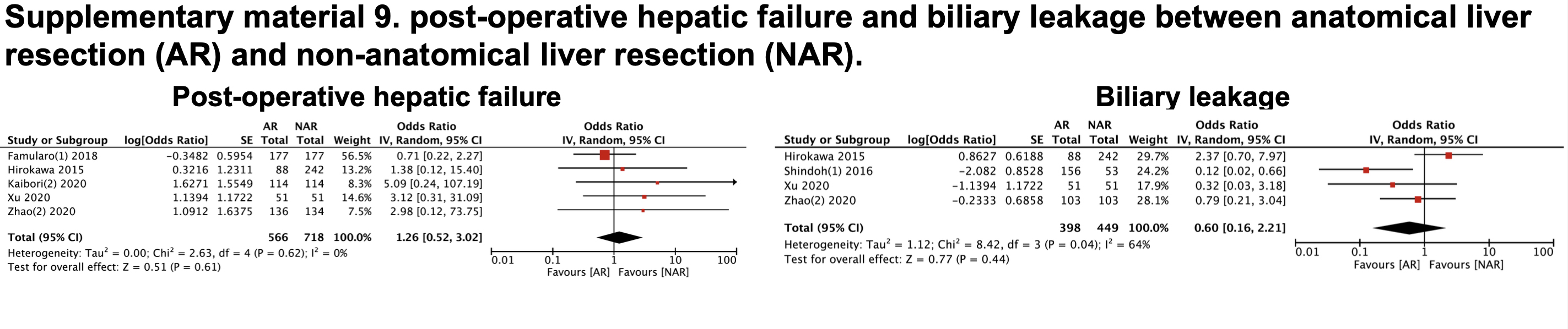

Supplement: SUPPLEMENTARY MATERIAL [file js9-109-2784-s004.jpg]

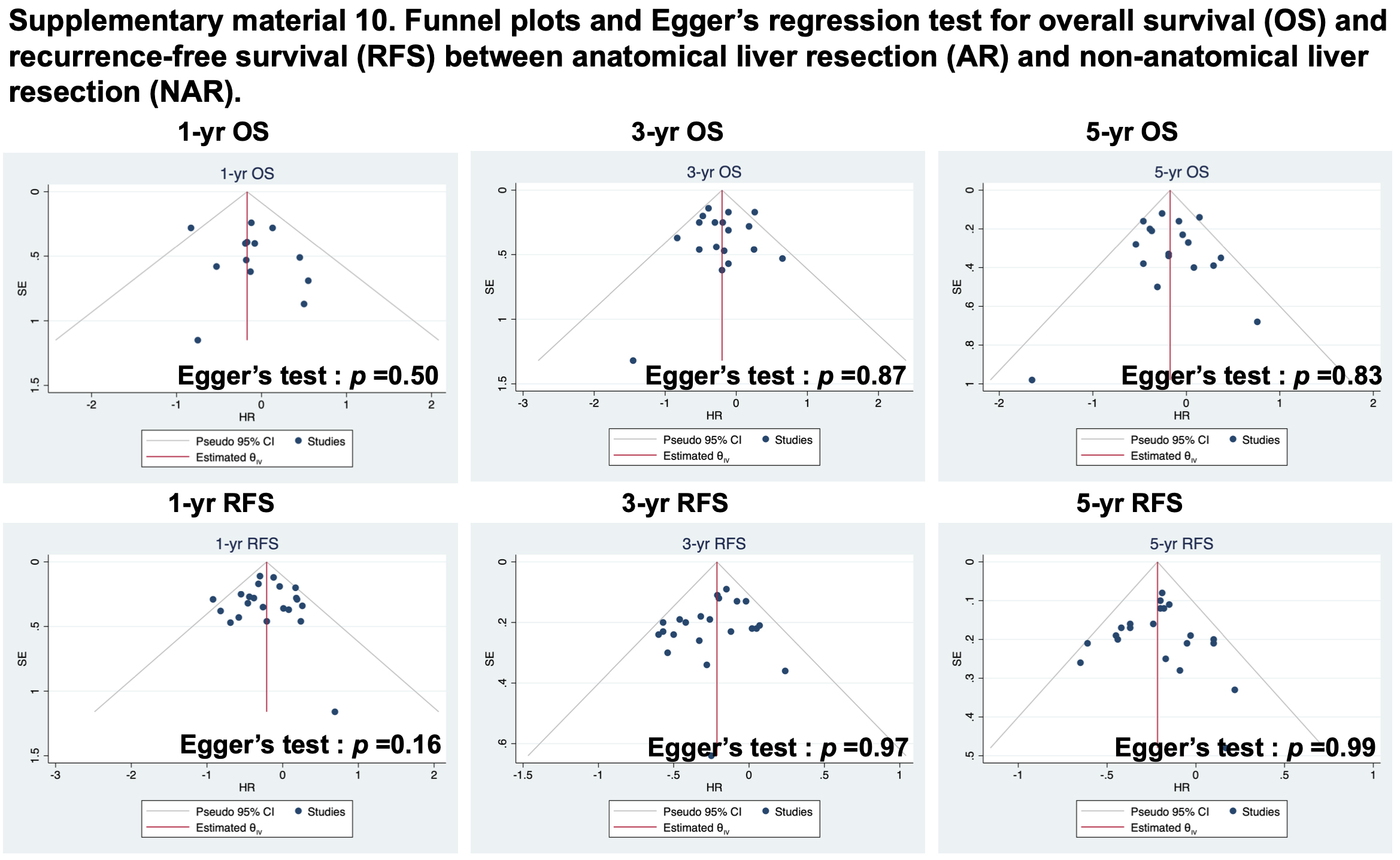

Supplement: SUPPLEMENTARY MATERIAL [file js9-109-2784-s005.jpg]

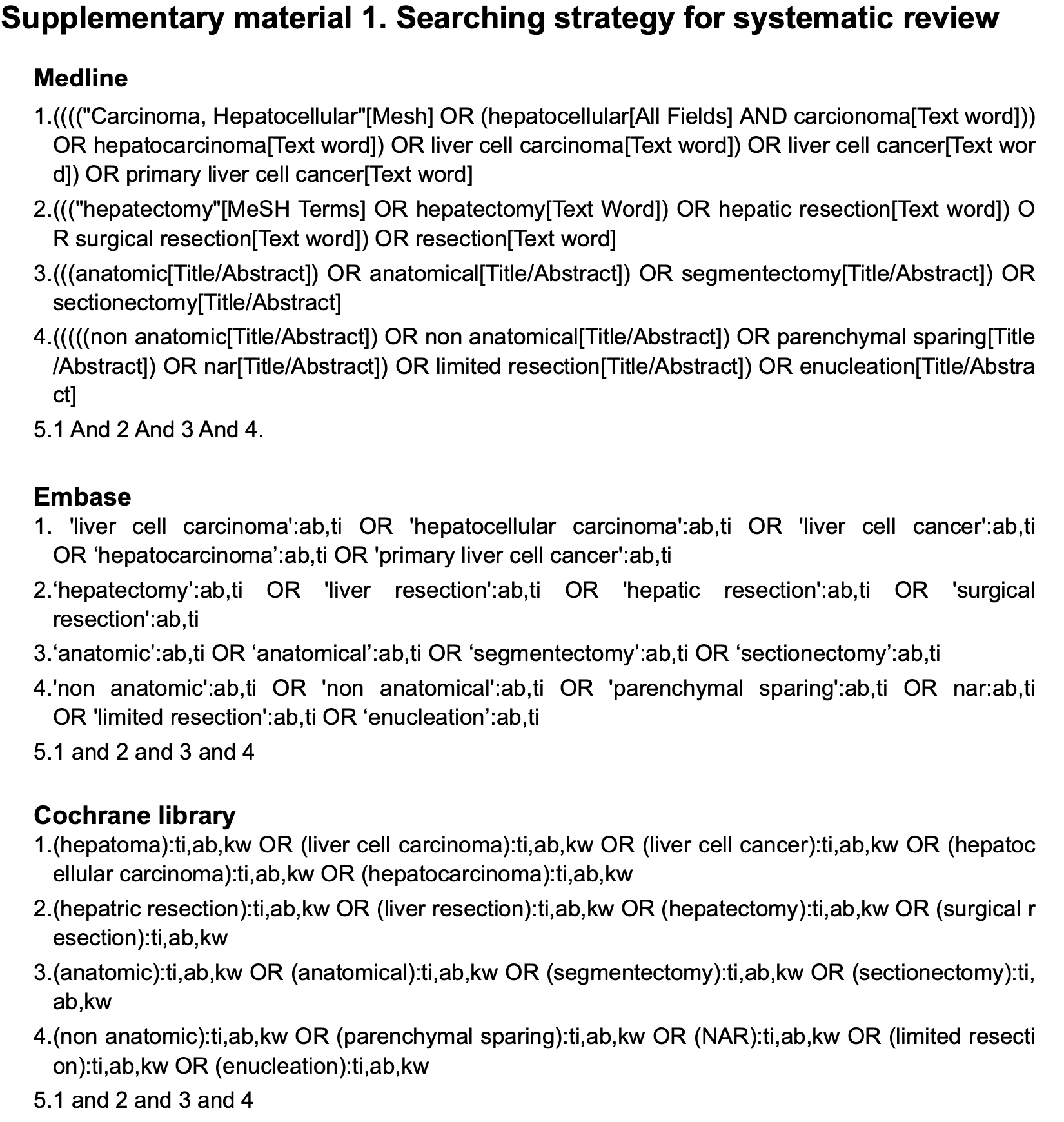

Supplement: SUPPLEMENTARY MATERIAL [file js9-109-2784-s008.jpg]

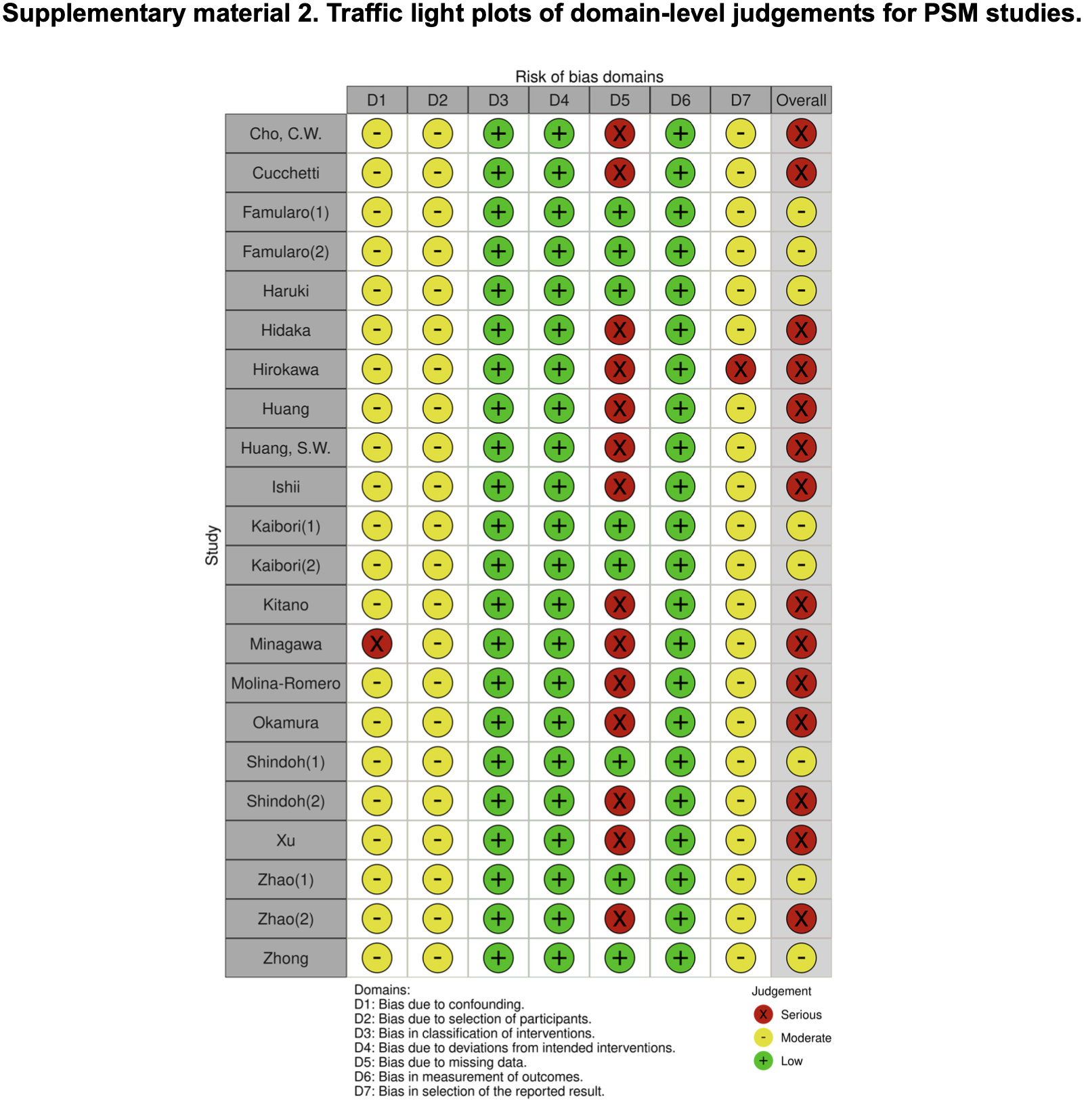

Supplement: SUPPLEMENTARY MATERIAL [file js9-109-2784-s009.jpg]

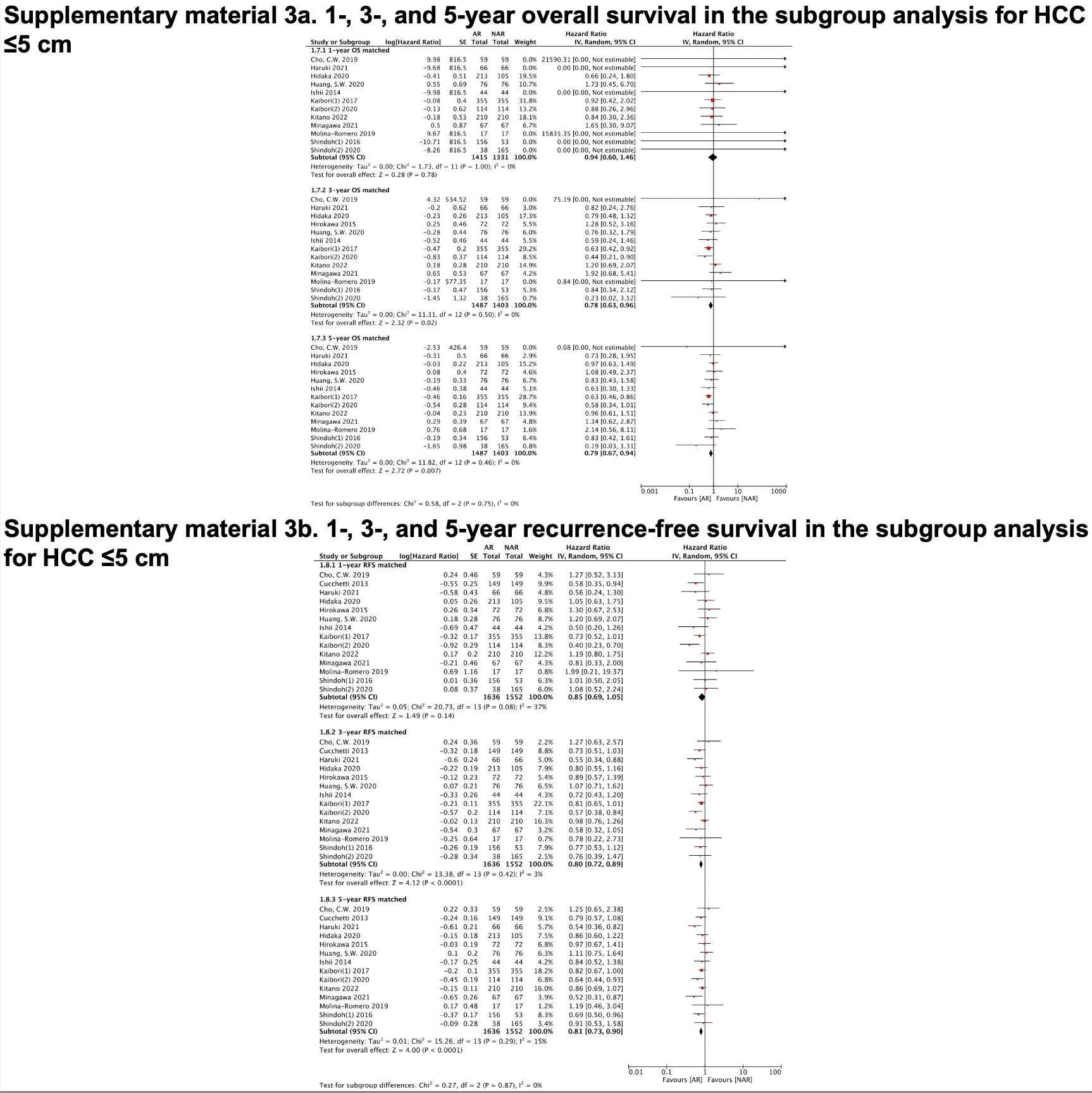

Supplement: SUPPLEMENTARY MATERIAL [file js9-109-2784-s010.jpg]

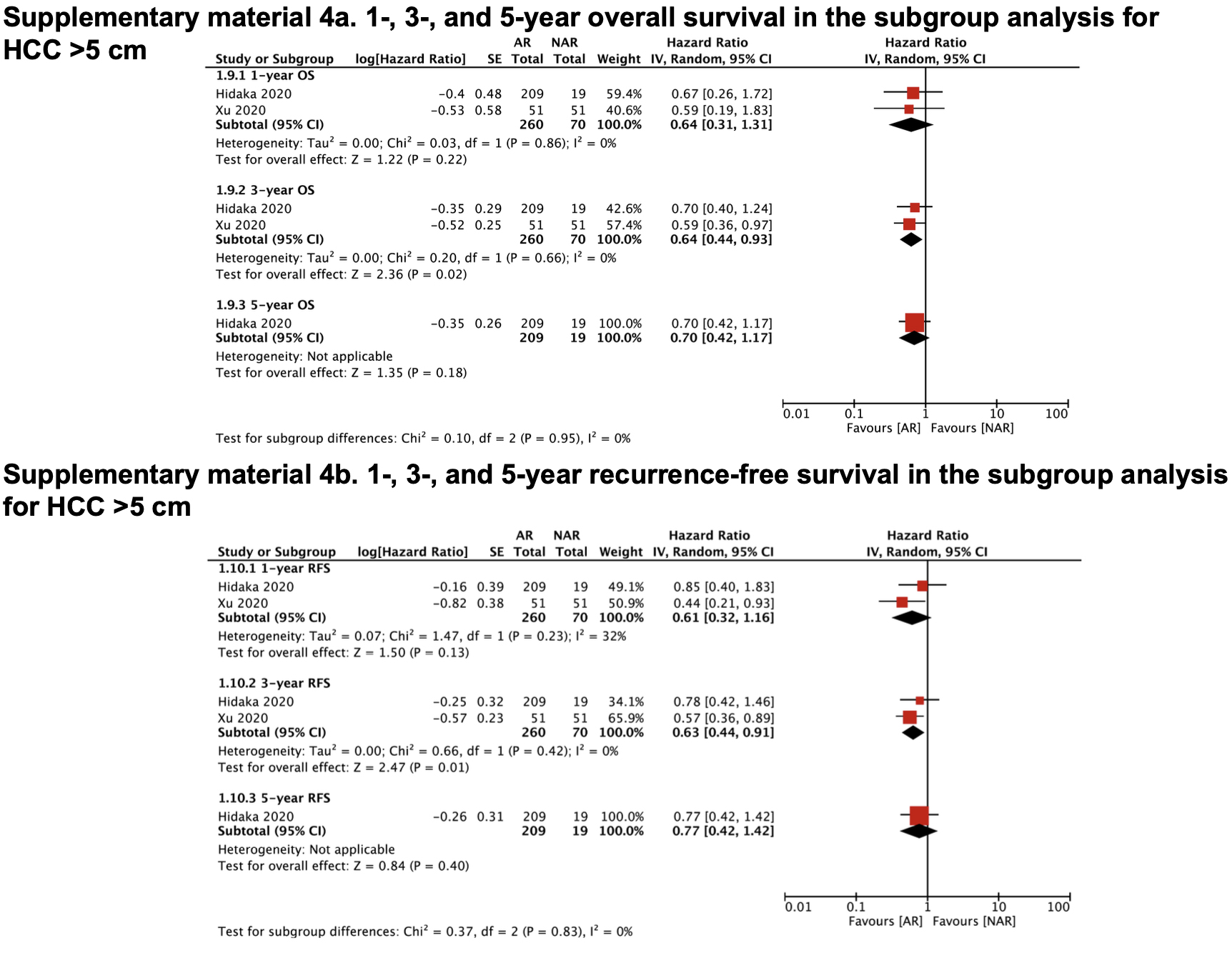

Supplement: SUPPLEMENTARY MATERIAL [file js9-109-2784-s011.jpg]

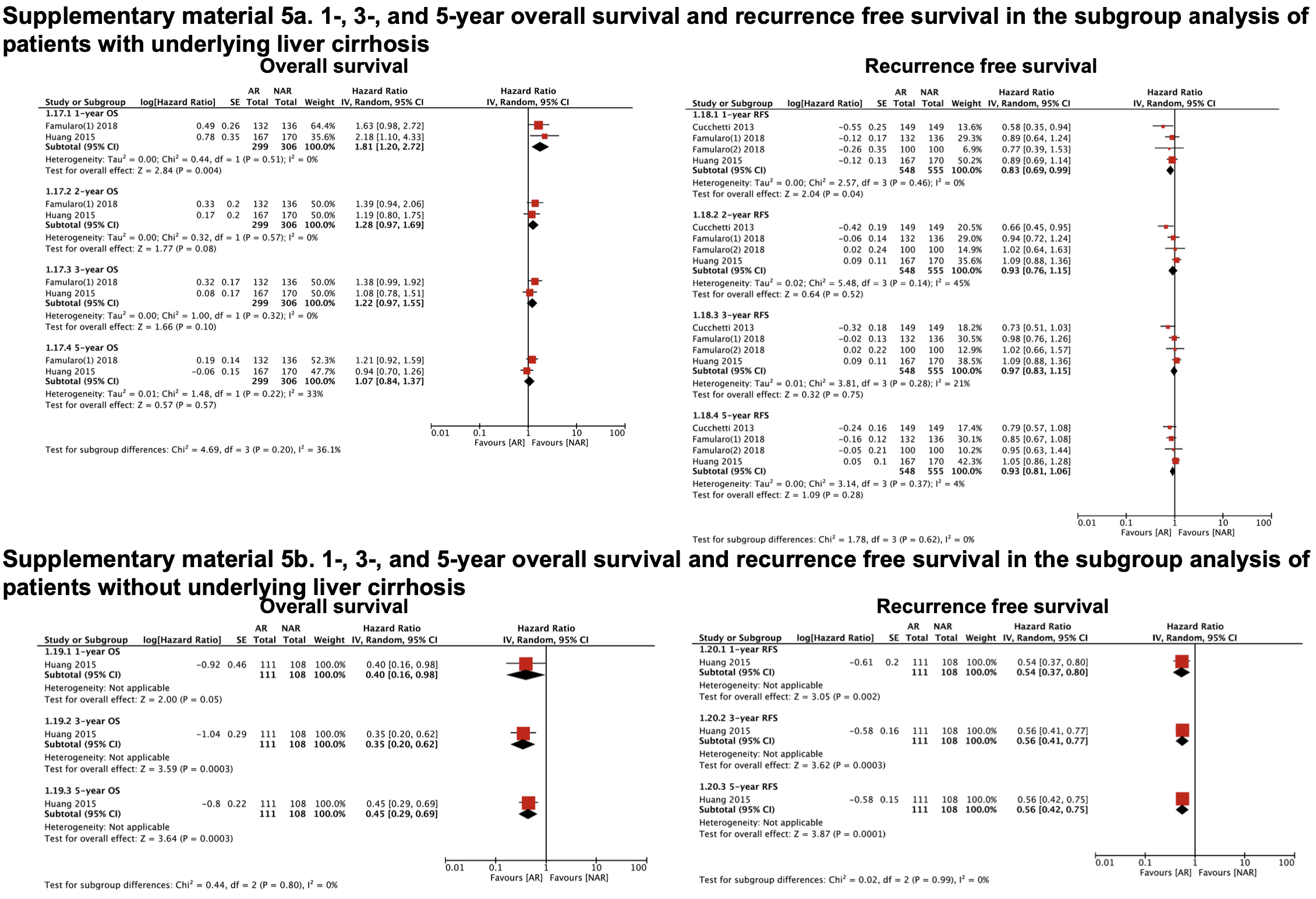

Supplement: SUPPLEMENTARY MATERIAL [file js9-109-2784-s012.jpg]

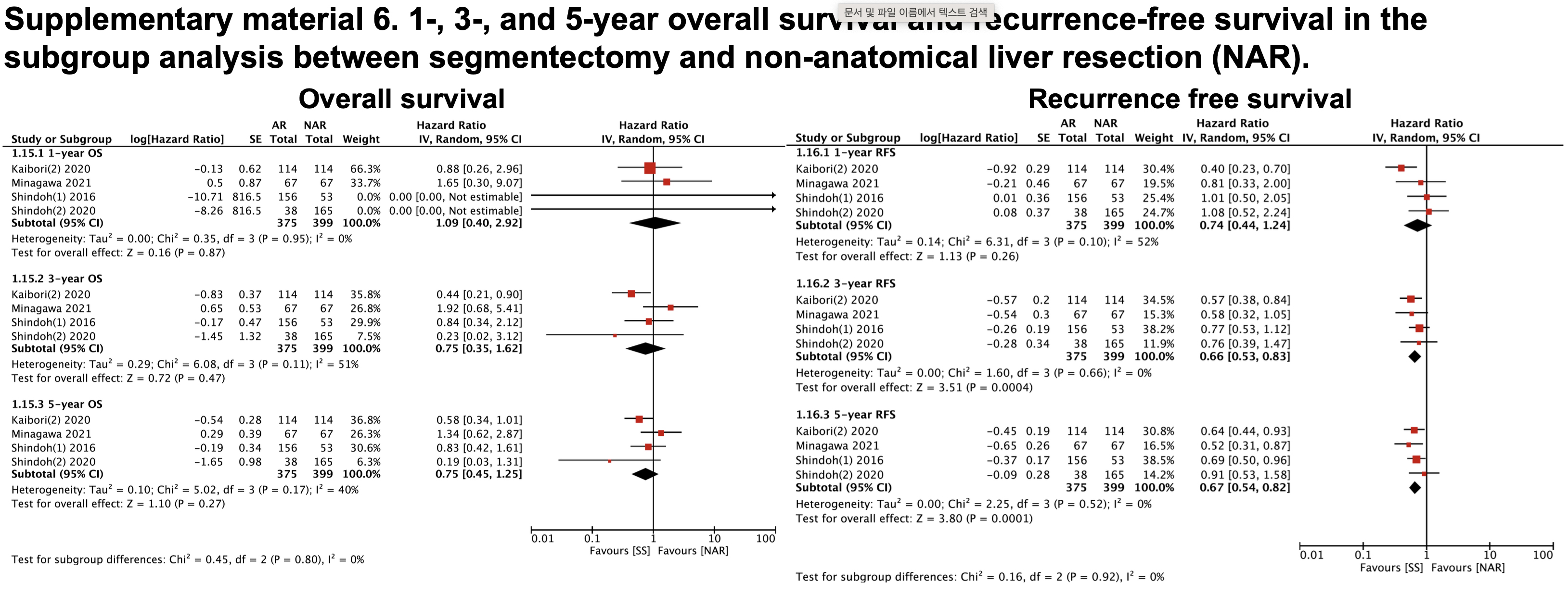

Supplement: SUPPLEMENTARY MATERIAL [file js9-109-2784-s013.jpg]
