# Supplementary material for: Effect of anatomical liver resection for hepatocellular carcinoma: a systematic review and meta-analysis
Source: Int J Surg. 2023 May 26;109(9):2784–93. doi: 10.1097/JS9.0000000000000503 (PMC10498869; doi:10.1097/JS9.0000000000000503)
Supplement: SUPPLEMENTARY MATERIAL [file js9-109-2784-s006.docx]

**Identification of studies via databases and registers**

Records removed *before screening*:

Duplicate records removed (n =96 )

Records identified from*:

Medline (n=149)

Embase (n=258)

Cochrane library (n=15)

**Identification**

Records screened

(n = 326)

Records excluded**

(n = 236)

Reports sought for retrieval

(n = 326)

Reports not retrieved

(n = 0)

Reports excluded (n=68):

- Non-propensity score matching studies (n=18)

- Randomized control trial (n=1)

- Propensity score matching study including patients with recurrent tumors (n=1)

- Propensity score matching study including patients with macrovascular invasion (n=1)

- Propensity score matching study including patients with macroscopic bile duct tumor thrombosis (n=1)

- Conference abstract (n=16)

- Studies including patients undertaken preoperative anti-cancer treatment (n=5)

- Studies including patients undertaken adjuvant therapies (n=3)

- Studies including patients with non-primary HCC (n=3)

- Review articles (n=17)

- Articles that could not find full text (n=2)

Reports assessed for eligibility

(n = 326)

**Screening**

Studies included in review

(n = )

Reports of included studies

(n = )

**Included**

*Consider, if feasible to do so, reporting the number of records identified from each database or register searched (rather than the total number across all databases/registers).

**If automation tools were used, indicate how many records were excluded by a human and how many were excluded by automation tools.

*From:*  Page MJ, McKenzie JE, Bossuyt PM, Boutron I, Hoffmann TC, Mulrow CD, et al. The PRISMA 2020 statement: an updated guideline for reporting systematic reviews. BMJ 2021;372:n71. doi: 10.1136/bmj.n71

For more information, visit: <http://www.prisma-statement.org/>
